# The effects of biological aging on global DNA methylation, histone modification, and epigenetic modifiers in the mouse germinal vesicle stage oocyte

**DOI:** 10.21451/1984-3143-AR2018-0087

**Published:** 2018-12-05

**Authors:** Kira Lynn Marshall, Juanbin Wang, Tieming Ji, Rocío Melissa Rivera

**Affiliations:** 1 Division of Animal Sciences.; 2 Department of Statistics.; 3 Reproductive Sciences, San Diego Zoo Global Institute for Conservation Research, San Pasqual Valley Rd.

**Keywords:** epigenome, Histone acetylation, Histone methylation, Methyl DNA binding domain proteins, DNA methyltransferase

## Abstract

A cultural trend in developed countries is favoring a delay in maternal age at first childbirth. In mammals fertility and chronological age show an inverse correlation. Oocyte quality is a contributing factor to this multifactorial phenomenon that may be influenced by age-related changes in the oocyte epigenome. Based on previous reports, we hypothesized that advanced maternal age would lead to alterations in the oocyte’s epigenome. We tested our hypothesis by determining protein levels of various epigenetic modifications and modifiers in fully-grown (≥70 µm), germinal vesicle (GV) stage oocytes of young (10-13 weeks) and aged (69-70 weeks) mice. Our results demonstrate a significant increase in protein amounts of the maintenance DNA methyltransferase DNMT1 (P = 0.003) and a trend toward increased global DNA methylation (P = 0.09) with advanced age. MeCP2, a methyl DNA binding domain protein, recognizes methylated DNA and induces chromatin compaction and silencing. We hypothesized that chromatin associated MeCP2 would be increased similarly to DNA methylation in oocytes of aged female mice. However, we detected a significant decrease (P = 0.0013) in protein abundance of MeCP2 between GV stage oocytes from young and aged females. Histone posttranslational modifications can also alter chromatin conformation. Di-methylation of H3K9 (H3K9me2) is associated with permissive heterochromatin while acetylation of H4K5 (H4K5ac) is associated with euchromatin. Our results indicate a trend toward decreasing H3K9me2 (P = 0.077) with advanced female age and no significant differences in levels of H4K5ac. These data demonstrate that physiologic aging affects the mouse oocyte epigenome and provide a better understanding of the mechanisms underlying the decrease in oocyte quality and reproductive potential of aged females.

## Introduction

In mammals, there is an inverse correlation between fertility and chronological age ( Broekmans et al., 2009 ; Qiao et al., 2014 ). Current cultural trends favor delaying child birth to later maternal ages, increasing the amount of conceptions occurring in females nearing the later part of their reproductive lifespan ( Ventura et al., 2012 ).

The age-related decline in mammalian female fertility is a multifactorial phenomenon greatly influenced by oocyte quantity ( Faddy, 2000 ) and quality ( Cohen et al., 1999 ), as well as lowered embryo development ( Talbert, 1971 ; van Kooij et al., 1996 ; Lopes et al., 2009 ). Contributing factors include decreased ooplasmic quality, mitochondrial defects, changes in chromatin structure, and meiotic defects ( Hassold and Hunt, 2001 ; Pan et al., 2008 ; Hornick et al., 2015 ). For example, in human and mouse the incidence of chromosomal abnormalities increases with female age due to non-disjunctions and other related errors in meiosis ( Plachot, 2001 ; Pan et al., 2008 ). Also, meiotic chromosomes isolated from oocytes of aged mice are more rigid than their young counterparts ( Hornick et al., 2015 ). Further, in mice, a useful animal model for the study of aging-related events ( Danilovich and Ram Sairam, 2006 ; Vanhooren and Libert, 2013 ), increased biological age is associated with alterations of the oocyte’s transcriptome ( Hamatani et al., 2004 ; Pan et al., 2008 ; Yue et al., 2012 ) and epigenome ( Akiyama et al., 2006 ; Manosalva and Gonzalez, 2009 ; Manosalva and González, 2010 ).

The epigenome includes covalent and non-covalent modifications to the DNA and associated histone proteins that control gene expression, chromatin structure, and genome stability ( Lande-Diner et al., 2007 ; Weber et al., 2007 ; Choy et al., 2010 ; Brenet et al., 2011 ). DNA methylation, the addition of a methyl group to cytosine residues primarily in a CpG context (5MeC), is associated with stable gene repression and involved in chromatin compaction ( Keshet et al., 1986 ; Pennings et al., 2005 ; Lande-Diner et al., 2007 ). This epigenetic modification is catalyzed by the *de novo* methyltransferases DNMT3a, DNMT3b, and the non-catalytic binding partner DNMT3L and maintained by DNMT1 ( Okano et al., 1999 ; Bestor, 2000 ). The repressive and compacting actions of DNA methylation are carried out by methyl CpG binding domain proteins (MBDs; e.g. MeCP2) that recognize methylated cytosines and recruit co-repressor complexes and histone modifiers that help in the silencing process ( Wakefield et al., 1999 ; Ohki et al., 2001 ; Zou et al., 2012 ).

Histones are small globular, alkaline proteins ranging in molecular weight between 11.3 to 21 kilodaltons ( DeLange et al., 1972 ; Elgin and Weintraub, 1975 ) that form an octamer around which 146 base pairs of DNA wrap to form a nucleosome. The amino terminus protrudes from the core structure and can be post-translationally modified. These modifications include acetylation (ac) and methylation (me) and each affects transcription and chromatin structure in a variety of ways by cross-talk with other epigenetic modifications (reviewed in: Kouzarides, 2007 ; Zentner and Henikoff, 2013 ). Histone acetylation occurs on lysine residues and this post-translational modification is associated with permissive euchromatin and active transcription ( Allfrey et al., 1964 ; Brownell et al., 1996 ; reviewed in: Zentner and Henikoff, 2013 ). Lysine residues can also be modified with the addition of one methyl group (mono-methylation; me1), two methyl groups (di-methylation; me2) or three methyl groups (tri-methylation; me3) ( DeLange and Smith, 1973 ). Histone methylation can be associated with both active and repressed transcription depending on the level, residue, and location in chromatin. For example, trimethylation of lysine 4 on histone 3 (H3K4me3) in promoter regions is associated with active transcription while H3K9me2 is found in facultative heterochromatin (silent DNA; Peters et al., 2003 ; Lachner et al., 2004 ; Bannister et al., 2005 ; Bernstein et al., 2005 ; Weber et al., 2007 ).

The epigenome of the oocyte and early embryo is very dynamic. Following recruitment, growing oocytes gradually acquire DNA methylation and histone modifications until reaching the fully grown, GV oocyte stage ( Monk et al., 1987 ; Sanford et al., 1987 ; Kafri et al., 1992 ; Lucifero et al., 2002 ; Lucifero et al., 2004 ; Hiura et al., 2006 ; Kageyama et al., 2007 ; Kota and Feil, 2010 ; Almamun, 2011 ; Hales et al., 2011 ; Plasschaert and Bartolomei, 2014 ). During progression from arrested GV stage oocyte to meiotic resumption in an MII oocyte, histones undergo global deacetylation and gene transcription ceases ( Hebbes et al., 1988 ; Bouniol-Baly et al., 1999 ; Kim et al., 2003 ; van den Berg et al., 2011 ).

Following fertilization, the epigenome is restructured to transition from the gametic genomes inherited from oocyte and sperm to a state allowing pluripotency and differentiation. The genome of the paternal pronucleus undergoes active DNA demethylation through the oxidation of 5MeC to 5-hydroxy-methyl cytosine by Ten-eleven translocation (TET3) in mice ( Rougier et al., 1998 ; Mayer et al., 2000 ; Oswald et al., 2000 ; Santos et al., 2002 ; Gu et al., 2011 ; Iqbal et al., 2011 ; Wossidlo et al., 2011 ; Peat et al., 2014 ). The maternal genome is demethylated passively through subsequent cleavage divisions. This is partially accomplished by nuclear exclusion of the oocyte-specific DNA methyltransferase (DNMT1o), except for transient entry during the 8-cell stage ( Carlson et al., 1992 ; Ratnam et al., 2002 ; Cirio et al., 2008 ; Hirasawa et al., 2008 ). Global DNA methylation is then re-established during the blastocyst stage ( Lepikhov et al., 2010 ; Hales et al., 2011 ).

Histone modifications are also asymmetric in the early embryo. The maternal pronucleus contains hypermethylated histones with both active (H3K4me1/2/3) and repressive (H3K9me1/2/3, H3K27me1/2/3, and H4K20me3) marks but incorporates acetylation at a slower rate than the paternal genome. The paternal genome decondenses and acquires maternal histones acetylated on H3K-8, -9, -14, and -18 and H4K-5, -12, and -16, and mono-methylated at H3K4, H3K9, and H3K27 to replace protamines and slowly acquires histone methylation H3K9me2. By the 4-cell stage, the pronuclei epigenomes become indistinguishable ( Adenot et al., 1997 ; Arney et al., 2002 ; Lepikhov and Walter, 2004 ; Santos et al., 2005 ; Lepikhov et al., 2010 ; Hales et al., 2011 ; Beaujean, 2014 ).

Previous preliminary research in our laboratory suggested an increased level of global DNA methylation in germinal vesicle (GV) stage oocytes from aged female mice when compared to their young counterparts. The discovery that physiologic aging decreases the elasticity of meiotic chromosomes (i.e. more rigid with increased age; Hornick et al., 2015 ) coupled with our preliminary results on increased levels of DNA methylation in oocytes from aged females led us to hypothesize that there is an increase in DNA methylation, MeCP2 and H3K9me2 and a decrease in H4K5ac in oocytes of aged females when compared to their young counterpart.

## Methods

### Mice

CF-1 (NSA; Harlan) mice were housed in groups on a 12 hour light/ dark cycle with access to food and water *ad libitum.* All animal procedures were performed as approved by the Institutional Animal Care and Use Committee of the University of Missouri.

### Oocyte collection and immunofluorescence

Fully grown GV stage oocytes (≥70µM) were isolated from ovaries of naturally cycling CF-1 female mice aged 10-13 weeks (young control) or 69-70 weeks (aged). We chose to examine GV stage oocytes, as errors in the epigenome at this phase would potentially affect the transcriptional library in the oocyte cytoplasm that must support the ovulated oocyte and early embryo. In addition, epigenetic errors in the GV oocyte could influence chromosome structure leading to later meiotic errors. Further, some ART procedures require utilizing pre-ovulatory oocytes and these procedures are more prevalent in aged females. While some epigenetic errors may be fixed during the reprogramming events of meiosis and development and thus not seen in MII stage oocytes, errors at the GV stage may affect transcript abundance and chromosome structure irreversibly.

Ovaries were placed in supplemented minimum essential medium (MEM) and oocytes were isolated by puncturing the ovary with a cluster of 27-gauge needles, allowing dissociation of oocytes. MEM (Sigma Aldrich, St. Louis, MO) was supplemented with 3 mg/ml polyvinylpyrrolidone (PVP), 25mM HEPES, 10mg gentamicin, and 0.9mM sodium pyruvate (pH ~7.3). Following isolation from the ovaries, all fully grown GV stage oocytes (≥70µM; measured with an eye piece micrometer) were transferred to fresh supplemented MEM. Cumulus cells were removed by incubating oocytes in MEM containing 1mg/ml type IV-S hyaluronidase (Sigma, St. Louis, MO) followed by thorough washing in MEM. Oocytes were incubated in Tyrode’s solution (pH 2.5; Sigma, St. Louis, MO) to remove the zona pellucida followed by thorough washing in MEM. Oocytes were then transferred into 1X phosphate buffered saline (PBS) containing 3mg/ml PVP (Sigma, St. Louis, MO).

### 5MeC

Oocytes were fixed in 4.0% paraformaldehyde (PFA; Electron Microscopy Sciences, Hatfield, PA) at room temperature for 15 minutes. Oocytes were then washed in PBS containing 0.05% Tween-20 (PBST20; Fisher Scientific, Waltham, MA) followed by plasma membrane permeabilization in 1X PBS containing 0.2% Triton X-100 (Electron Microscopy Sciences, Hatfield, PA) for 30 minutes. DNA was denatured in 2N HCl for 30 minutes followed by neutralization in 100mM Tris HCl, pH 8.5 for 10 minutes and washed thoroughly in PBST20.

Oocytes were incubated in blocking solution (PBST20 with 1% BSA) overnight at 4^o^ C in a humidified chamber. Oocytes were then incubated with a primary antibody against 5MeC (mouse monoclonal Calbiochem NA81, EMD Biosciences, Inc., La Jolla, CA; 1:500 dilution) for one hour at room temperature in a humidified chamber. Primary antibody specificity was previously validated by other groups ( Rougier et al., 1998 ; Mayer et al., 2000 ; Barton et al., 2001 ; Masala et al., 2017 ; Salmina et al., 2017 ). Following washes in blocking solution, samples were stained with secondary antibody (Alexafluor 555 rabbit anti-mouse, Invitrogen, Grand Island, NY; 1:200 dilution) for 30 minutes at room temperature in a humidified chamber in the dark followed by extensive washing in blocking solution. Controls included oocytes without exposure to primary antibody, oocytes without exposure to secondary antibody, and oocytes without exposure to both antibodies (i.e. blocking solution alone was used for incubations).

### DNMT1, MeCP2, H3K9me2, H4K5ac

Oocytes were fixed in 3.7% PFA at room temperature for 20 minutes. Oocytes were then washed in PBST20 followed by plasma membrane permeabilization in 1X PBS containing 0.2% Triton X-100 for 15 minutes at room temperature followed by thorough washing in PBST20. Oocytes were incubated in blocking solution for one hour at room temperature in a humidified chamber. Oocytes were then incubated in primary antibodies against MeCP2 (rabbit monoclonal, Cell Signaling #3456, Danvers, MA; 1:50 dilution), dimethylated lysine 9 of histone 3 (rabbit polyclonal, H3K9me2; Cell Signaling #9753; 1:100 dilution), acetylated lysine 5 of histone 4 (H4K5ac; rabbit polyclonal, Abcam ab61236, Cambridge, MA; 1:500 dilution), or DNMT1 (rabbit polyclonal, Sigma-Aldrich D4567, St. Louis, MO; 1:500 dilution) overnight at 4^o^C in a humidified chamber. Primary antibody specificity was determined by the manufacturers (DNMT1, MeCP2, H3K9me2, H4K5ac). Following washes in blocking solution, samples were stained with secondary antibody (Alexaflour 555 rabbit anti-mouse or goat anti-rabbit, Invitrogen, Grand Island, NY; 1:200 dilution) for 30 minutes at room temperature in a humidified chamber in the dark followed by extensive washing in blocking solution. Controls included oocytes without exposure to primary antibody, oocytes without exposure to secondary antibody, and oocytes without exposure to both antibodies (i.e. blocking solution alone was used for incubations).

### DNA staining and mounting

DNA was stained with 2µM YoYo1 Iodide (excitation/emission peaks = 458 nm/564 nm; Invitrogen, Grand Island, NY) diluted in blocking solution for 30 minutes at room temperature in a humidified chamber in the dark and washed in blocking solution. Alternatively, DNA was stained with 1.2 μM DRAQ 7 (excitation/ emission peaks = 633nm/695nm; Abcam, Cambridge, MA) diluted in blocking solution for one hour at room temperature in a humidified chamber in the dark, transferring to a fresh drop every 20 minutes. The oocytes were then washed in blocking solution. It should be noted that both DNA stains were used interchangeably through the experiments as their only purpose was to visualize DNA. No measurements were taken of the fluorescence emitted from these dyes.

Oocytes were incubated in 75% Vectashield Hardset Mounting Medium (Vector Laboratories, Burlingame, CA) diluted in blocking solution and 100% Vectashield. Oocytes were mounted in 5µL Vectashield on a #1 coverslip that had been dabbed on all four corners with Vaseline containing glass beads ≥106 µm (Sigma-Aldrich, St. Louis, MO). Coverslips were sealed to the slide with clear nail polish to prevent drying.

### Image acquisition and analysis

Digital sections of individual oocytes were recorded using an SP2 Confocal/ Multiphoton Microscope (Leica, Bannockburn, IL) and water immersion objective (63X) with lasers at 488 nm and 543 nm wavelengths. For detection, the laser power was set to the level at which the young control oocytes had the strongest fluorescence intensity but showed no saturation signal. Confocal settings remained constant within each technical replicate. Confocal images displaying the largest cross section of the nucleolus were used for analysis. A region of interest was drawn around the oocyte for DNMT1 or the GV for all others and the sum of fluorescence in all selected pixels (integrated density; (IntDen)) was measured in arbitrary units (AU) using Metamorph (Molecular Devices, Sunnyvale, CA). For these measurements, a threshold of 4 AU was applied, so only pixels above 4 AU were included in the measurement. This threshold was chosen because it corresponded to the highest fluorescence measure in areas of background. Sample size and distribution by mouse and trial is shown in Table 1

**Table 1:** Oocyte and trial distribution for young and aged female mice.

	5MeC				MeCP2				DNMT1				H3K9me2				H4K5ac			
	young		aged		young		aged		young		aged		young		aged		young		aged	
Trial	oocytes	mice	oocytes	mice	oocytes	mice	oocytes	mice	oocytes	mice	oocytes	mice	oocytes	mice	oocytes	mice	oocytes	mice	oocytes	mice
1	20	2	7	2	15	2	7	3	5	2	1	1	22	2	11	4	21	2	7	2
2	30	3	11	3	12	1	4	3	31	3	21	7	20	2	7	4	21	2	1	1
3	23	2	14	5	21	2	12	5	23	2	27	7	20	2	8	3	18	2	8	4
4	20	2	11	4	27	3	13	5					17	2	26	5	27	3	13	8
5	18	2	4	3																
6	22	2	6	2																
Total	133	13	53	19	75	8	36	16	59	7	49	15	79	8	52	16	87	9	29	13

### Statistical analysis

#### Immunofluorescence

Average integrated densities of oocytes from all female mice were recorded for each of the five conditions. Observations were then modeled by Gaussian linear mixed models. Specifically, age was modeled as a fixed effect. Mice and trials were modeled as random effects to capture correlations among oocytes that came from the same mouse and mice that came from the same trial. In addition, data for H4K5ac were modeled under the original scale, and data for H3K9me2, DNMT1, 5MeC, and MeCP2 were log transformed prior to modeling to maintain Gaussian model assumptions. The SAS software tool (Cary, NC) was used to implement our models. By testing on the fixed age effect, we drew conclusions on whether integrated densities of oocytes on average are significantly different between the young and the aged groups. To evaluate changes in variance with age, variance of oocyte integrated densities was calculated for each female and log transformed to maintain Gaussian model assumptions. All females with only one oocyte observation were removed from the variance analysis since variance cannot be assessed with one observation. Variances were modeled by Gaussian linear mixed models with age as a fixed effect and trial as a random effect. Model assumptions were examined by checking residual plots, and statistical conclusions were drawn by testing age effect on the change of variance between the young and the aged groups.

## Results

### DNA methylation and associated proteins

**Figure 1:**
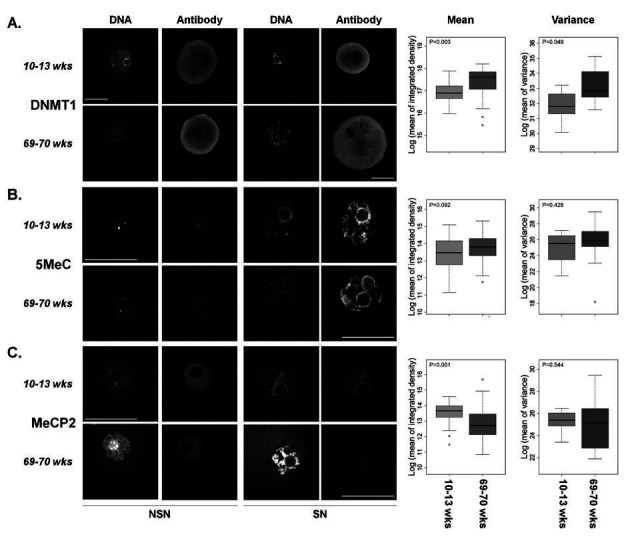
Immunofluorescent localization of DNA modifications/modifiers in fully-grown GV stage oocytes of young and aged female mice. Left panels: Shown are single confocal sections of the nucleus (GV; 5MeC, MeCP2) or oocyte (DNMT1). Left-most column are examples of oocytes with a non-surrounded nucleolus conformation (NSN = relaxed chromatin) and the right most column are micrographs of the surrounded nucleolus conformation (SN = contracted chromatin). No differentiation was made between these conformations for further analyses. Confocal settings were the same within each technical replicate. Stains used interchangeably to visualize DNA were YoYo1 Iodide or DRAQ7. Scale bar = 50 µm. Right panels: Shown are boxplots of the integrated density for each antibody (i.e. sum of all pixels above background; left) and variance of mean integrated density (right). The p-values are presented in the top left corner of each boxplot. Young mice = 10-13 weeks. Aged mice = 69-70 weeks. A. Data for DNMT1 from three trials representing 59 oocytes from 7 young mice and 49 oocytes from 15 aged mice. B. Data for 5MeC from six trials representing 133 oocytes from 13 young mice and 53 oocytes from 19 aged mice. C. Data for MeCP2 from four trials representing 75 oocytes from 8 young mice and 36 oocytes from 16 aged mice.

We observed a trend towards increased levels of global DNA methylation (P = 0.09; Figure 1B ) in oocytes of aged females when compared to their young counterparts. We also identified a significant increase in levels of DNMT1 protein (maintenance DNA methyltransferase) with increased maternal age (P = 0.003; Figure 1A ). Lastly, we found a significant decrease in levels of chromatin associated MeCP2 with aging (P = 0.0013; Figure 1C ).

### Histone post-translational modifications

**Figure 2:**
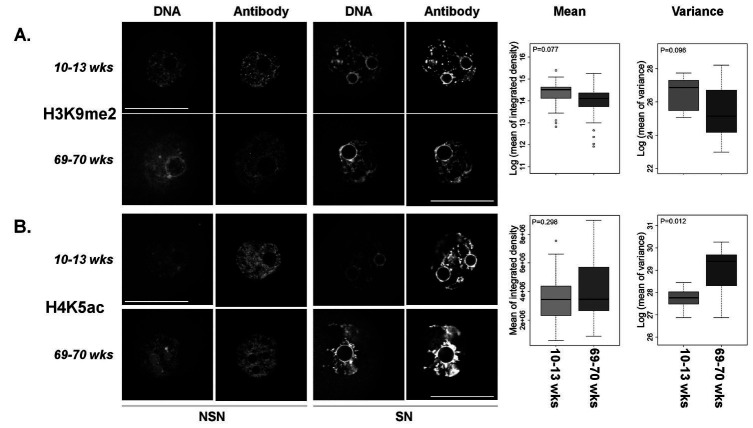
Immunofluorescent localization of histone modifications in fully-grown GV stage oocytes of young and aged female mice. Left panels: Shown are single confocal sections of the nucleus (GV). Left-most column are examples of oocytes with a non-surrounded nucleolus conformation (NSN = relaxed chromatin) and the right most column are micrographs of the surrounded nucleolus conformation (SN = contracted chromatin). No differentiation was made between these conformations for further analyses. Confocal settings were the same within each technical replicate. Stains used interchangeably to visualize DNA were YoYo1 Iodide or DRAQ7. Scale bar = 50 µm. Right panels: Shown are boxplots of the integrated density for each antibody (i.e. sum of all pixels above background; left) and variance of mean integrated density (right). The p-values are presented in the top left corner of each boxplot. Young mice = 10-13 weeks. Aged mice = 69-70 weeks. A. Data for H3K9me2 from four trials representing 79 oocytes from 8 young mice and 52 oocytes from 16 aged mice. B. Data for H4K5ac from four trials representing 87 oocytes from 9 young mice and 29 oocytes from 13 aged mice.

**Figure 3:**
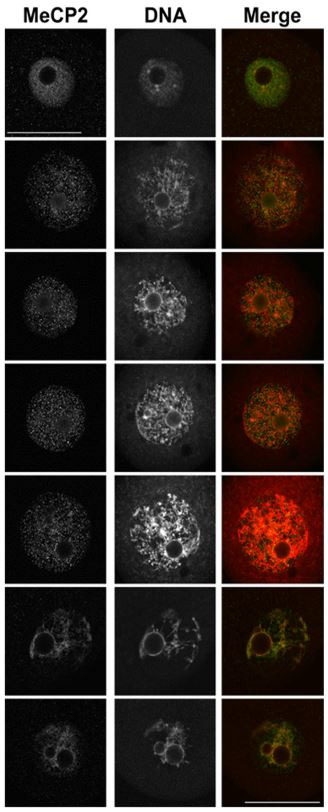
Confocal images for MeCP2 staining. Shown are single confocal sections of the nucleus (GV) displaying MeCP2 (*left*), DNA (*middle)*, or a merged image of both stains (*right).* In the merged image, green represents MeCP2, red corresponds to DNA and yellow indicates areas of colocalization. Confocal settings were the same within each technical replicate. DNA stain = YoYo1 Iodide or DRAQ7. Note the lack of MeCP2 localization in chromocenters and nucleolar rings. Scale bar = 50 µm.

Levels of H3K9me2 tended to decrease as a function of biological age (P = 0.077; Figure 2A ) in fully-grown, GV stage oocytes, however, no difference was observed in levels of global H4K5ac between oocytes from young and aged mice (P = 0.3; Figure 3B ).

### Age-dependent variance of epigenetic modifiers

During the course of analysis, it became evident that there was a high degree of variation in protein levels as measured by staining in the oocytes from aged females. In order to test if this was different, we compared average variance in staining intensity between oocytes from aged and young mice. While there were no significant age-associated differences in the variance of 5MeC and MeCP2 staining (P = 0.43 and 0.54, respectively; Figure 1B and C right panels), the variance of staining intensity of DNMT1 and H4K5ac were significantly increased (P = 0.049 and 0.012, respectively; Figure 1A , 2B right panels) in oocytes from aged mice. H3K9me2 staining showed a trend toward a decreased variance in oocytes from aged females (P = 0.096; Figure 2A right panel).

### Immunolocalization of MeCP2 in GV stage oocytes

**Supplemental Figure 1:**
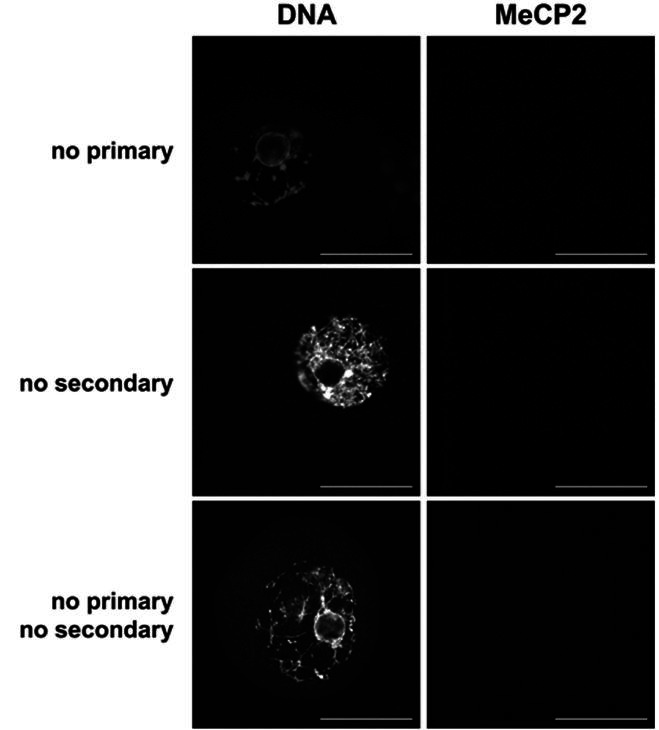
Immunofluorescent no antibody control images for MeCP2 staining. Shown are single confocal sections of the nucleus (GV) without exposure to primary antibody (*top* ), secondary antibody (*middle*), or either antibody (*bottom *). Confocal settings were the same within each technical replicate. DNA stain = YoYo1 Iodide. Scale bar = 50 µm.

Previous studies have shown conflicting results as to the presence of MeCP2 in mouse oocytes at both the mRNA and protein level ( Kantor et al., 2003 ; Ruddock-D'Cruz et al., 2008 ; Song et al., 2014 ). Our data support the presence of MeCP2 protein in fully-grown GV stage oocytes ( Figure 3 and Supplemental Figure 1 ).

### Chromatin configuration in oocytes from aged females

**Figure 4:**
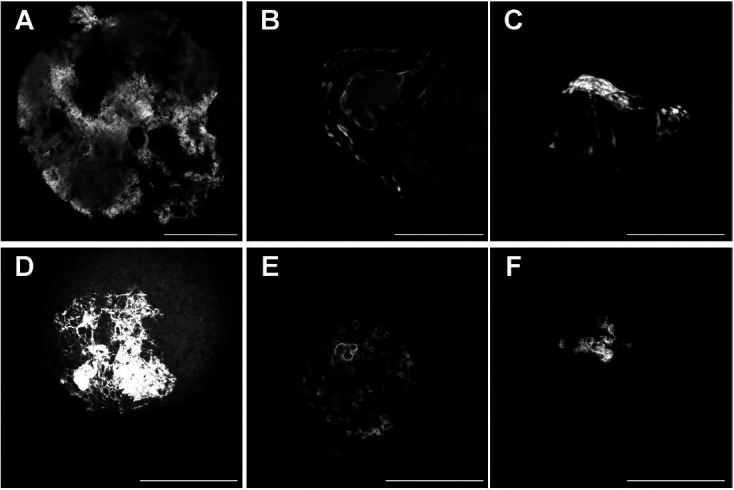
Immunofluorescent images of abnormal oocytes in fully-grown germinal vesicle-stage oocytes. Shown are single confocal sections of germinal vesicle (GV) or oocytes. A. Misshapen and torn oocyte from aged female stained for DNMT1. B-D. GV of oocytes from aged mice displaying a distorted nuclear structure stained for H3K9me2 (B and C) and DNA (D). E-F. GV of oocytes displaying DNA circles from a young mouse and aged mouse stained for H3K9me2 and H4K5ac, respectively. Scale bar = 50 µm.

Aged oocytes were often larger than their young counterparts and were more susceptible to tearing ( Figure 4A ), indicating the potential that these larger oocytes may be more fragile. Even though the possibility exists that oocyte tearing may have been the result of pressure being applied by the coverslip, we do not believe this is the case as the glass beads used for mounting were ≥106 µm, which should have prevented the oocytes from being crushed. Many of the aged oocytes also exhibited a distorted GV structure, sometimes even lacking a well-defined nucleolus ( Figure 4B-D ). In addition, a variety of abnormal chromatin states were observed ( Figure 4 ). Another unique pattern of chromatin distribution seen in oocytes from eight aged mice and one young mouse ( Figure 4E-F ) was a large number of DNA circles.

## Discussion

Multiple lines of evidence demonstrating meiotic abnormalities and aberrant transcription associated with physiologic aging ( Hassold and Hunt, 2001 ; Hamatani et al., 2004 ; Pan et al., 2008 ; Yue et al., 2012 ) point to the epigenome as a contributor to age-related changes in oocyte quality and reproductive potential. DNA methylation and histone modifications interact to regulate gene expression and chromatin structure in a variety of ways.

DNA methylation is an epigenetic modification associated with repression of transcription and formation and stabilization of heterochromatin ( Keshet et al., 1986 ; Watt and Molloy, 1988 ; Boyes and Bird, 1991 ; Lande-Diner et al., 2007 ). In our study, we were interested in expanding our previously observed preliminary result in which an increase in DNA methylation with advanced age was apparent. Here, we identified a trend towards increased levels of global DNA methylation in oocytes of aged females when compared to their young counterparts. Our DNA methylation results indicate a tendency opposite the age-related patterns previously observed by Yue and colleagues ( Yue et al., 2012 ). Several reasons may exist for the difference in levels of 5MeC between the study by Yue and ours. First, the type of oocyte used for analysis differed between studies (Yue = meiosis II (MII) stage oocytes; Marshall = GV stage oocytes). It is possible that an age-related change in these modifiers is not evident until after ovulation has occurred. Second, the mouse strain used; the present study used CF-1 mice while the Yue study used Kunming white mice. Third, we used aged females which were almost twice as old as those used by Yue (69-70 weeks vs. 35-40 weeks). Fourth, our study used hormonally unstimulated females while the Yue study used gonadotropin-primed females. This latter point is relevant, as we ( Huffman et al., 2015 ) and others ( Shi and Haaf, 2002 ; Sato et al., 2007 ; Market-Velker et al., 2010 ) have shown that superovulation can affect the levels of DNA methylation in oocytes and developing embryos. Because of the role of DNA methylation in transcriptional repression and chromatin compaction, an increase in global DNA methylation with increased female age is a likely contributor to oocyte transcriptome alterations previously reported ( Hamatani et al., 2004 ; Pan et al., 2008 ; Yue et al., 2012 ), possibly affecting developmental potential of the embryo. In addition, the observed increased DNA methylation likely plays a role in decreased chromosome elasticity seen by Hornick and colleagues ( Hornick et al., 2015 ) by increasing chromatin compaction. Future experiments to determine downstream effects of increased DNA methylation at the GV stage could help to elucidate the role DNA methylation and aging plays in fertility and embryonic development.

DNA methylation is catalyzed and maintained by the DNMTs. The main role of DNMT1 is maintenance of DNA methylation during DNA replication ( Hermann et al., 2004 ; Sharif et al., 2007 ). DNMT1 has also been shown to increase the efficiency of *de novo* methylation, a process which involves *de novo* hemimethylation by DNMT3a followed by the copying of the DNA methylation to the unmethylated strand by DNMT1 ( Fatemi et al., 2002 ). We observed an age-dependent increase in protein levels of DNMT1. Again, our results regarding DNMT1 protein levels conflict with findings from Yue ( Yue et al., 2012 ). As a methyltransferase, increased DNMT1 stored in the oocyte could lead to a more significant increase in DNA methylation during embryo development. For example, a study evaluating imprinted gene methylation and expression in embryonic stem cells found overexpression of DNMT1 was associated with increased DNA methylation of *Igf2* and *H19*, leading to biallelic expression or downregulation respectively ( Biniszkiewicz et al., 2002 ). As these genes are important to growth during development, we postulate increased DNMT1 in oocytes of aged females is a likely contributor to decreased reproductive success, though experiments to determine the mechanisms are needed.

The remodeling and repressive actions of DNA methylation are accomplished through recruitment of MBDs ( Boyes and Bird, 1991 ; Zou et al., 2012 ). MeCP2 is an MBD that recognizes and binds to methylated CpG pairs and interacts with co-repressors and histone deacetylases ( Jones et al., 1998 ; Wakefield et al., 1999 ) to induce chromatin reorganization and transcriptional silencing ( Jones et al., 1998 ; Nan et al., 1998 ; Georgel et al., 2003 ). MeCP2 can also block transcription by interacting directly with transcription factors and preventing formation of pre-initiation complexes ( Kaludov and Wolffe, 2000 ). We expected to see higher levels of MeCP2 associated with advanced female age in concert with the observed increase in DNA methylation. However, we found a significant decrease in levels of MeCP2. The decrease in levels of MeCP2 protein in relation to an increased female age has not previously been reported. While chromatin compaction is an established role for MeCP2, a report indicates DNA methylation is not necessary for MeCP2 to induce alterations in chromatin structure ( Baubec et al., 2013 ). This could at least partially explain why MeCP2 did not mimic the age-related changes in DNA methylation. It is also possible that MeCP2 is not the prominently active MBD in the oocyte and the increase in chromatin rigidity found by Hornick and others ( Hornick et al., 2015 ) is mediated by the action of DNA methylation interaction with other MBDs aside from MeCP2.

MBDs act as transcriptional repressors through chromatin compaction in concert with other chromatin remodelers including histone deacetylases (HDACs) ( Jones et al., 1998 ). Previous reports demonstrate that levels of *Hdac2* are decreased in MII stage oocytes with increased female age ( Hamatani et al., 2004 ), similar to the pattern seen here with MeCP2. These parallel decreases in chromatin remodelers responsible for repression may explain a portion of the alterations in levels of gene expression that coincide with female aging ( Hamatani et al., 2004 ; Pan et al., 2005 ; Yue et al., 2012 ). Because of known functions of MeCP2 in chromatin contraction and repression of transcription ( Jones et al., 1998 ; Georgel et al., 2003 ) and research demonstrating the localization of MeCP2 to areas of heterochromatin in somatic cells ( Marchi et al., 2007 ), we hypothesized that levels of MeCP2 would concentrate in areas of heterochromatin such as the nucleolar ring and chromocenters ( Marchi et al., 2007 ; Singleton et al., 2011 ) but instead it appeared diffuse throughout areas of euchromatin with a punctate pattern that was evident in both age groups evaluated. A mosaic localization of MeCP2 has also been shown in the murine developing brain ( Kishi and Macklis, 2004 ) where a more diffuse pattern, like the one observed in our studies, was evident in the nuclei of undeveloped tissues and the more typical heterochromatic localization was seen with neuronal maturation and differentiation. Perhaps MeCP2 localizes to heterochromatic regions after fertilization in the mouse although no data exist to validate this possibility.

The methylome works in concert with the histone epigenome. As our preliminary observations indicated increased DNA methylation, we hypothesized a corresponding increase in repressive histone modifications. We focused our efforts on H3K9me2 as this histone post-translational modification is associated with facultative (permissive) heterochromatin ( Lachner et al., 2001 ; Peters et al., 2001 ; Peters et al., 2003 ). During early embryogenesis, the maternal pronucleus retains H3K9 dimethylation and maternal histones possessing this mark replace protamines on the paternal genome ( Arney et al., 2002 ; Lepikhov and Walter, 2004 ; Santos et al., 2005 ). H3K9me2 rich regions bind PCG7 (also called Stella or DPPA3) to protect DNA methylation in these regions from active demethylation by TET3 ( Nakamura et al., 2012 ). With such a critical role in global DNA methylation levels, alterations to levels of H3K9me2 in the oocyte could have future impacts on resulting embryo DNA methylation and demethylation. Contrary to our hypothesis, levels of H3K9me2 tended to decrease as a function of biological aging. This age-related decrease in H3K9me2 supports findings from other laboratories showing an age-related decrease in GV and MII oocytes from 11 month old mice ( Manosalva and González, 2010 ). Histone methyltransferases G9a and SETDB1 are responsible for demethylating H3K9 and are involved in chromosome stability and segregation ( Schultz et al., 2002 ; Tachibana et al., 2002 ; Tachibana et al., 2007 ). Kondo and colleagues ( Kondo et al., 2008 ) demonstrated centromere disruption, shortened telomere length, and chromosome instability following knockdown of *G9a.* The role of these demethylases in regulating chromatin structure and meiotic integrity lead us to postulate that decreased H3K9me2 in oocytes from aged females may contribute to the increase in meiotic errors seen with advanced maternal age.

Opposite to H3K9me2, histone acetylation is deposited in areas of euchromatin and is associated with active transcription ( Allfrey et al., 1964 ; Brownell et al., 1996 ; reviewed in: Zentner and Henikoff, 2013 ). Lysine 5 of histone 4 is the last residue on this histone to acquire acetylation and the first residue from which acetylation is removed, so H4K5ac is considered the mark of hyperacetylation ( Turner, 2008 ). Because it is first residue to be deacetylated, we reasoned this would be the most susceptible to epigenetic errors associated with aging. We expected to see a decrease in the presence of this mark with increased female age however, no difference was observed in levels of global H4K5ac between oocytes from young and aged mice. These data are in accordance with data from Manosalva and Gonzalez ( Manosalva and Gonzalez, 2009 ) who showed comparable levels of acetylation in GV stage oocytes from aged and young female mice. While we saw no change in the level of this modification, previous studies have demonstrated age-related changes in acetylation of other residues of histone 4 such as H4K12ac and HK16ac which are lower in GV stage oocytes from aged mice ( Manosalva and Gonzalez, 2009 ).

During the course of analysis, it became evident that there was a high degree of variation in protein levels as measured by staining in the oocytes from aged females. In order to test if this was different, we compared average variance in staining intensity between oocytes from aged and young mice. While there were no significant age-associated differences in the variance of 5MeC and MeCP2 staining, the variance of staining intensity of DNMT1 and H4K5ac were significantly increased in oocytes from aged mice. H3K9me2 staining showed a trend toward a decreased variance in oocytes from aged females. The increased variability in levels of H4K5ac and DNMT1 could indicate a greater sensitivity of these epigenetic components to increased female age.

Many of the aged oocytes exhibited a distorted GV structure, sometimes even lacking a well-defined nucleolus. In addition, a variety of abnormal chromatin states were observed in these oocytes. Another unique pattern of chromatin distribution seen in oocytes from eight aged mice and one young mouse was a large number of DNA circles. DNA circles have been documented in yeast ( Sinclair and Guarente, 1997 ). These circles were determined to be fragmented nucleoli and be associated with aging yeast cells. It is possible that these circles indicate DNA fragmentation in damaged oocytes or demonstrate oocytes destined to undergo apoptosis. However, no information is available regarding the presence of this phenomenon in mammalian cells.

In conclusion, we observed alterations in the epigenome of the GV stage oocyte as a result of increased female age. Levels of DNMT1 were significantly increased and 5MeC displayed a trend of increased levels in oocytes from aged female mice. In contrast, MeCP2 presence was decreased, along with a trend toward decreased levels of H3K9me2. The function of these differences requires more study to elucidate the effect these epigenetic alterations are imparting on oocytes from females of advanced age.
